# Sirt1 interaction with active Smad2 modulates transforming growth factor-β regulated transcription

**DOI:** 10.1186/s12964-017-0205-y

**Published:** 2017-11-29

**Authors:** Eva María García-Vizcaíno, Sergio Liarte, José Luis Alonso-Romero, Francisco José Nicolás

**Affiliations:** 1 Laboratorio de Oncología Molecular y TGFβ, Instituto Murciano de Investigaciones Biosanitarias Arrixaca, El Palmar, Murcia, Spain; 2 Servicio de Oncología, Hospital Clínico Universitario Virgen de la Arrixaca, El Palmar, Murcia, Spain

**Keywords:** Sirt1, TGFβ-signaling, Tumor transformation, Protein interaction, Gene transcription regulation

## Abstract

**Background:**

The simplicity of Transforming Growth Factor ß (TGFβ) signaling pathway, linear and non-amplified, hardly sustains its variety of responses. This is often justified by the complex regulation showed by Smad proteins, TGFβ signaling intracellular transducers, object of post-translational modifications that modulate TGFβ-dependent transcription. Protein acetylation is emerging as a compelling mechanism affecting the activities of significant transcription factors, including p53, FOXO or NF-kB. Smad proteins might be controlled by this mechanism, implying that accessory factors capable of altering Smads-transcriptional complexes acetylation status and hence regulate TGFβ responses remain to be identified. Understanding this interaction may help in the assessment of TGFβ signaling outcomes, extending from healthy physiology to pathological conditions and cancer.

**Methods:**

A two-hybrid chimera interacting system allowed to identify Sirt1, a NAD+ dependent type III histone deacetylase, as a novel Smad2 interactor. Several well stablished cellular models were applied to characterize this interaction by means of co-immunoprecipitation of tagged proteins and immuno-fluorescence staining. The occurrence of the interaction at Smad2 driven transcriptomic complexes was studied by means of DNA-pull-down and chromatin immunoprecipitation (ChIP), while its effects were assessed by protein over-expression and siRNA applied into a TGFβ-dependent reporter gene assay.

**Results:**

The interaction was confirmed and observed to be enhanced upon Smad2 acetylation, a known feature of active and nuclear Smad2. However, Sirt1 did not play a major role in Smad2 deacetylation. Anti-Sirt1 ChIP showed increased recovery of promoter regions corresponding to Smad2-driven genes after TGFβ-stimulation, while its occurrence at Smad2-dependent transcriptomic complexes on DNA was found to effectively modulate gene expression.

**Conclusions:**

Sirt1 presence on Smad2-driven TGFβ-dependent regulatory elements was detected and found to increase after TGFβ treatment. Moreover, Sirt1 overexpression resulted in a decrease of the activity of a Smad2-driven TGFβ-dependent reporter gene, while Sirt1 interference increased its activity. This would confirm the relevance of the discovered Sirt1-Smad2 interaction for the regulation of TGFβ-dependent gene transcription.

**Electronic supplementary material:**

The online version of this article (10.1186/s12964-017-0205-y) contains supplementary material, which is available to authorized users.

## Background

TGFβ regulates numerous cellular responses involved in cell proliferation and differentiation, embryonic development, wound healing, angiogenesis and apoptosis [1, 2]. Upon TGFβ binding, TGFβ receptor (TGFR) sub-units I and II interact and form a heterotetramer with an active cytosolic serine/threonine kinase domain. TGFβ signaling mediators, the receptor-regulated (R-Smads), Smad2 and Smad3, are then phosphorylated to become active and mobilized into the nucleus. Once there, they interact with common-mediator (co-Smad), Smad4, in order to conform protein complexes that execute transcription regulation [3]. However, the simplicity of the linear and non-amplified TGFβ pathway does not justify the wide diversity of biological responses observed [4]. These responses, which are cell-type specific and dependent on cell physiological status, often depict a controversial role for TGFβ in pathological conditions, especially in cancer where suppressive and pro-oncogenic roles have been demonstrated for this cytokine [5, 6]. This implies a complex regulation of TGFβ signaling [7], and suggests that unidentified factors may regulate Smad activities and thus modulate the TGFβ responses.

Post-translational modifications of the factors involved in TGFβ signaling appears as a main system to functionally modulate the TGFβ transducing pathway. On top of the triggering phosphorylation events, several works have shown the capital influence that changes to the acetylation status of Smads have on their activities [8]. TGFβ stimulation has been shown to promote CBP/p300- and PCAF-dependent acetylation of active Smad2, thereby promoting Smad2 nuclear accumulation by decreased nuclear export [9]. Likely, Smad3 has been shown to be subjected to acetylation, also promoting its nuclear accumulation [10]. Interestingly, these acetylations targeting both R-Smads have been shown to result in increased TGFβ-dependent transcription [9–11]. Also, Smads deacetylation has been shown to play an essential role in the regulation of TGFβ signaling. TGFβ stimulation has been shown to trigger Smad7 deacetylation, prior to the ubiquitination and proteasome degradation of this inhibitory Smad (i-Smad) which blocks Smad2 access to the active TGFβ receptor [12]. Deacetylation of Smad7 is mediated by several histone deacetylases (HDACs), mainly HDAC1, HDAC5 and HDAC6 [13]. Interestingly, Sirt1, a type III HDAC, has also been described to be involved in the inactivation of Smad7 [14].

HDACs are receiving more and more attention, as their ability to perform activities on substrates different from histones has been revealed. Sirt1 is a NAD+ dependent HDAC, which was described initially to deacetylate nuclear histones H1, H2 and H4 [15]. However, reports now indicate that Sirt1 acts on significant non-histone nuclear proteins, such as p53, FOXO, E2F1 or NF-kB, as well as cytosolic proteins [16–18]. Sirt1 actions are highly dependent on the cellular context and its activity has been involved in a wide range of biological processes and pathologies, ranging from modulating energy metabolism and metabolic syndromes, cellular senescence and ageing, development and degenerative processes, as well as immune disorders and inflammatory disease [19–21]. Notably, in addition to its demonstrated role on Smad7, Sirt1 has been recently shown to deacetylate Smad3 in a process linked to decreased Smad3 transcriptional output [22]. Intriguingly, similar to above mentioned role of TGFβ, Sirt1 also displays a paradoxical role in cancer [17, 20, 21, 23]. Histological studies have revealed increased and decreased expression patterns for Sirt1, depending on cancer type and/or stage [23, 24]. More importantly, Sirt1 activities had been lately shown to have an impact in epithelial-mesenchymal transition (EMT) process [25–27], a known hallmark of TGFβ deregulation [28].

In this work, using a two-hybrid chimera interacting system, we report the specific interaction of Sirt1 with Smad2. Subsequent analyses confirmed and further characterized this interaction, revealing the presence of Sirt1 in transcriptomic complexes and its relevance for the modulation of Smad2-driven gene transcription. Due to the complexity of TGFβ signaling, we postulate that this interaction and its role in Smad2-dependent signaling, may help in better understanding the complex regulation of TGFβ signaling and its diversity of outcomes. The implications towards Smads physiology and gene expression regulation are discussed.

## Methods

### Cyto Trap® yeast two hybrid assay

A screening for proteins interacting with Smad2 was performed by using the Cyto Trap® Yeast Two Hybrid System (Cyto Trap® X Premade Libraries, Stratagene, Agilent technologies, Santa Clara, CA, USA) following manufacturer’s instructions. Briefly, Smad2 was cloned into provided pSos vector to generate a bait construct. Smad2 fusion products were then validated against Smad4-pMyr target fusion product. Once validated, a commercial Human lung plasmid cDNA library (Stratagene, Agilent technologies, Santa Clara, CA, USA) was cloned into the pMyr vector and target constructs were applied for screening using the *S. cerevisiae* strain Cdc25H (α) provided with the kit. Within the system, just bait-target interactions allow for Ras activation and yeast growth at 37 °C temperature, which is used as selective condition.

### Cell culture and treatments

Human hepatocellular carcinoma cells (Hep3B) [11], a kind gift of Dr. Isabel Fabregat, were grown in MEM Eagle-EBSS (EMEM) (LONZA, Basel, Switzerland). Human spontaneously immortalized Keratinocyte cell line (HaCaT)(kindly given by Dr. Caroline S. Hill) [29], Human embryonic kidney cells (HEK293T) [30] and mouse fibroblasts (NIH-3 T3) [31] were grown in Dulbecco’s Modified Eagle Medium (DMEM) (LONZA, Basel, Switzerland). Both media were supplemented with 10% Fetal Bovine Serum (FBS) (GIBCO, Thermo Fisher Scientific, Waltham, MA, USA), 1% Penicillin/Streptomycin and 1% L-Glutamine (LONZA, Basel, Switzerland). HaCaT-EGFP-Smad2 cells [32], a kind gift from Dr. Caroline S. Hill, were grown in DMEM media supplemented, as above, and kept in selection with 0.5 mg/ml of G418 (Acros Organics, Thermo Fisher Scientific, Waltham, MA, USA).

Stimulation with TGFβ was performed for the indicated times with 2 ng/ml recombinant TGFβ1, that was reconstituted following manufacturer instructions (PEPROTECH, PeproTech, Rocky Hill, NJ, USA). Treatment with the TGFβ type I receptor inhibitor SB-431542 10 μM or the type I and II deacetylase inhibitor Trichostatin-A (TSA) 2.5 μM (both from Sigma-Aldrich, St Louis, MO, USA) is indicated where used.

### Transfection and expression of epitope tagged proteins

Transfection of HEK293T, Hep3B and NIH-3 T3 cell lines was performed using Lipofectamine (Invitrogen, Thermo Fisher Scientific, Waltham, MA, USA) according to manufacturer’s instructions. Human Smad2 was amplified by PCR and cloned into the expression vector pEF-Flag [31], kindly gifted by Dr. Caroline S. Hill. Human Sirt1 obtained by CytoTrap Two Hybrid system was directly cloned by enzymatic digestion into pEF-HA [33]. Two different Smad2 mutants (K19R, K20R, K39R and K19Q, K20Q, K39Q) were obtained over pEF-Flag-Smad2 construct using QuikChange Site-Directed Mutagenesis Kit (Invitrogen, Thermo Fisher Scientific, Waltham, MA, USA). Same strategy was used to obtain the Sirt1H363Y mutant [34]. HA-Mixer, Flag-Mixer, Flag-FoxH1 and pEF-XC, and the reporters ARE-Luc, and DE-Luc were all a kind gift of Dr. Caroline S. Hill [31, 33]. After transfection, cells were allowed to express different proteins for 24 h before experiments were performed. pEF-XC was used to normalize concentration of DNA transfected in luciferase assay. Transfection efficiency was assessed using pRL-TK (Promega, Madison, WI, USA). Dual-Luciferase Reporter Assay System was used following manufacturer instructions (Promega, Madison, WI, USA).

### Bacterial expression of proteins and protein pull-down assays

Smads-GST fusion proteins were obtained by cloning different fragments of Smad2 and Smad3 from pEF-vectors into pGEX-4 T-1 (GE Healthcare Life Science, Pittsburgh, PA, USA). Obtained plasmids were transferred into BL21 *E. coli* strain, and selected colonies were sequenced, tested for the expression and expected size of GST-fusion proteins. Purification of fusion products was performed using Glutathione-Sepharose™ 4B (GE Healthcare Life Science, Pittsburgh, PA, USA) and reduced glutathione (Sigma-Aldrich, St Louis, MO, USA) elution method. To obtain 6xHis tagged versions of Sirt1, corresponding cDNA were PCR-cloned into pQE-70 vector (Qiagen, Venlo, The Netherlands). Resulting plasmids were transferred into M15[pREP4] *E. coli* strain. Selected colonies were sequenced and tested for the expression of expected protein-6xHis fusions. Purification of the different 6xHis-proteins was done using QIAexpress Ni-NTA Fast Start kit (Qiagen, Venlo, The Netherlands) following instructions from the manufacturer.

For protein pull-down assays, 4 μg of GST-fusion proteins were mixed with 20 μl (10–20 μg) of previously blocked 6xHis resin-coupled fusion proteins in 400 μl of 50 mM HEPES pH 7.2, 300 mM NaCl, 1 mM EDTA, 1.2 mM MgCl_2_, 1% Triton X-100, 10% Glycerol (all from Sigma-Aldrich, St Louis, MO, USA) supplemented with 10 mM NaButyrate, 1 mM DTT, 25 mM NaF, 25 mM β-glycerophosphate, 1:100 phosphatase inhibitors (I and II), 1:100 protease inhibitors (all from Sigma-Aldrich, St Louis, MO, USA) and BSA 0.2 mg/ml (Santa Cruz Biotechnology, Heidelberg, Germany). Protein mix was incubated for 2 h at 4 °C and later, resin was washed three times with previously described buffer. When pull-down assay was performed with Flag-Smad2, the expressed protein was affinity-purified from transfected HEK293T cells using M2 anti-Flag antibody (Sigma-Aldrich, St Louis, MO, USA) and eluted by competition with 400 μg/ml of FLAG peptide (Sigma-Aldrich, St Louis, MO, USA) in 20 mM HEPES, 100 mM KCl, 0.5 mM EDTA and 10% Glycerol (all from Sigma-Aldrich, St Louis, MO, USA). Affinity purified Flag-Smad2 was added to the pull-down mixture containing Sirt1-6xHis or different fragments of it coupled to 6xHis epitope. This mix was incubated for 2 h at 4 °C and then resin was washed with buffer previously described. In all cases, samples were analyzed by western blot.

### Immunoprecipitation, western blot, immunostaining and antibodies

Total protein extracts were obtained by lysis of harvested cells using lysis buffer: 50 mM HEPES pH 7.2, 150 mM NaCl, 1 mM EDTA, 1.2 mM MgCl_2_, 1% Triton X-100, 10% Glycerol (all from Sigma-Aldrich, St Louis, MO, USA), supplemented with 10 mM NaButyrate, 1 mM DTT, 25 mM NaF, 25 mM β-glycerophosphate, 1:100 phosphatase inhibitors (I and II) and 1:100 protease inhibitors (all from Sigma-Aldrich, St Louis, MO, USA). Nuclear extracts were obtained as described elsewhere [32, 35]. For immunoprecipitation, 500 ng of appropriate antibody was added to 400 μg of protein total extract and incubated for 1 to 2 h at 4 °C. Antibodies were purified using A and G protein coupled sepharose (GE Healthcare Life Science, Barcelona, Spain) previously blocked with BSA 0.2 mg/ml (Santa Cruz Biotechnology, Heidelberg, Germany) in lysis buffer. In all cases, samples were analyzed by SDS-PAGE followed by western blot with appropriate antibodies. Quantification of the experimental data western blot signal was performed using Quantity One software by Bio-Rad (Hercules, CA, USA). Fold increase was calculated as the ratio between final value (B) and the original value (A): (B/A). At this point, sometimes, we observed that Sirt1 has a tendency to bind to nonspecific-antibody conjugated Sepharose, different buffers did not resolved that issue. We quantified that value (background value). To find out the Sirt1 specific binding value, the experimental value was decreased with the background value, then, the fold increase was calculated and represented in the figures. Immunostaining techniques were performed as described elsewhere [36, 37]. Images were taken using a confocal microscope LSM 510 META (ZEISS, Jena, Germany). Co-localization analyses were performed using the co-localization module of the ZEN software (ZEISS, Jena, Germany).

The following commercial antibodies were used: phospho-Smad2, phospho-Smad3, acetyl-Lys (all from Cell Signaling Technology, Danvers, MA, USA); Smad4, GST, Smad2 and Sirt1 (all from Santa Cruz Biotechnology, Heidelberg, Germany); Flag M2, ß-Actin (all from Sigma-Aldrich, St Louis, MO, USA); Smad2/3 (BD Transduction Laboratories, Beckton Dickinson, Franklin Lakes, NJ, USA); HA-Peroxidase (ROCHE, Sigma-Aldrich, St Louis, MO, USA); Sirt1 (Millipore, Darmstadt, Germany); Penta-His (Qiagen, Venlo, The Netherlands).

### DNA pull-down assay

DNA pull-down assays were performed as previously described [38]. Briefly, for each condition, 5 μg of 5′-biotinylated double-stranded oligonucleotides corresponding to the wild-type DE of the *goosecoid* promoter [33] (5’CTAGC CAT**TA AT**CAG **ATTA**A CGGTG AGCA**A TTA**GA CTAG3’ [TAAT motifs are in bold type]), or a version mutated for the Mixer/Smad binding sequence (5’CTAGC CA**G**T**C** ATCAG A**G**T**C**A CGGTG AGCAA **G**T**C**GA CTAG3’ [mutated nucleotides are in bold type]), or corresponding to the wild-type *c-jun SBR* of the *c-JUN* promoter [39] (5′GGAGG TGCGC GGAGT CAGGC **AGACA GACAG AC**ACA GCCAG CCAGC CAGGT CGGCA 3′ [the AGAC motifs are in bold type]), or a version mutated in the Smad3/Smad4 binding sites and flanking CCAG repeats (5′ GGAGG TGCGC GGAGT CAGGC **ATATA TATAT ATA**CA G**CATG CATGC ATG**GT CGGCA 3′ [mutated motifs are in bold type]) were bound to 30 μl of NeutrAvidin®-Agarose Resin (Thermo Fisher Scientific, Waltham, MA, USA). DNA pull-down was performed using 300 μg nuclear extract, supplemented with 5 μg of Sirt1-6xHis when indicated, in buffer containing 20 mM HEPES pH 7.6, 20% Glycerol, 140 mM NaCl, 1.5 mM MgCl_2_, 0.2 mM EDTA, 10% Triton X-100, supplemented with protease inhibitor 1:1000 (all from Sigma-Aldrich, St Louis, MO, USA) and containing 20 μg of non-biotinylated mutant oligonucleotides to reduce nonspecific binding. Assays were incubated overnight and after washing, bound proteins were detected by western blot. The c-Jun promoter DNA pull-down assay was performed similarly using oligonucleotides already described [39].

### Protein expression interference plasmids

Tet-inducible pTER siRNA vector was a kind gift from Dr. Hans Clevers, and cloning was performed as previously described [40]. Briefly, the Sirt1 siRNA Tet-inducible expression vectors pTER-siRNA1 and pTER-siRNA2 were generated by cloning the following annealed oligonucleotides into pTER. siRNA1: 5’GATCC CCGAT GAAGT TGACC TCCTC ATTCA AGAGA TGAGG AGGTC AACTT CATCT TTTTG GAAA3’ and 5’AGCTT TTCCA AAAAG ATGAA GTTGA CCTCC TCATC TCTTG AATGA GGAGG TCAAC TTCAT CGGG3’; this generates an siRNA directed against the sequence GATGAAGTTGACCTCCTCA, corresponding to nucleotides 1291 to 1309 of Sirt1 relative to the ATG. siRNA2: 5’GATCC CCCCT TCTGT TCGGT GATGA ATTCA AGAGA TTCAT CACCG AACAG AAGGT TTTTG GAAA3’ and 5’AGCTT TTCCA AAAAC CTTCT GTTCG GTGAT GAATC TCTTG AATTC ATCAC CGAAC AGAAG GGGG3’; this generates an siRNA directed against the sequence CCTTCTGTTCGGTGATGAA, corresponding to nucleotides 435 to 453 of Sirt1 relative to the ATG. To distinguish transfected cells and measure siRNAs efficiency, GFP cDNA was cloned together with a CMV promoter from plasmid pEGFP-C1 into pTER-siRNA1 and pTER-siRNA2. GFP positive cells were isolated by means of high-speed cell sorting (Beckman Coulter, CA, USA) 24 h after transfection.

### Chromatin immuno-precipitation and quantitative PCR

Chromatin immuno-precipitation (ChIP) was performed using the MAGnify reagent kit (Life Technologies, CA, USA) following manufacturer’s instructions. Briefly, semi-confluent HaCaT cells were exchanged to OPTIMEM medium (GIBCO, Thermo Fisher Scientific, Waltham, MA, USA) and cultivated overnight. Then, cells were treated with 2 ng/ml TGFβ for 1.5 h. Afterwards cells were washed twice with PBS and fixed using formaldehyde to a final concentration of 10% at room temperature (RT) for 8 min. The cross-link reaction was stopped by incubating cells for 8 min at RT in glycine 0.125 M. Cells were washed twice and scraped using cold PBS. The cell pellet was washed with PBS and re-suspended to determine total cell number. Then, cells were lysed using the supplied buffer supplemented with protease inhibitor cocktail for 10 min at RT. Cell lysates were sonicated in a Bioruptor™ Next Gen (Diagenode, Seraing, Ougrée, Belgium) to obtain 350–650-bp fragments. Chromatin corresponding to 0.75 million cells per condition and antibody were diluted using supplied IP buffer to a final volume of 100 μl. Dynabeads A/G beads provided in the kit were incubated for 1 h at 4 °C with supplied control IgG or the following specific antibodies: RNAPol II (Covance, NJ, USA); phospho-Smad2/3 (Abcam, Cambridge, UK); Sirt1 (Millipore, MA, USA). Diluted chromatin was incubated for 16 h at 4 °C with bead-combined-antibodies to capture the immune complexes. Magnetic beads were collected and washed following manufacturer’s directions and chromatin extracts were treated for reverse-crosslinking using supplied reagents. Samples of extract taken prior to IP were processed in parallel with the IPs and considered as inputs. Real time q-PCR was performed in triplicate for each IP reaction using respective primer set: PAI-SBR Fwd 5′cagccagacaaggttgttgacaca3′; PAI-SBR Rvs 5′ccagccacgtgattgtctaggttt3′; PAI-TSS Fwd 5′acacacacacacacacatgcctca3′; PAI-TSS Rvs 5′ccagatgtgggcaggaaatagatg3′; JUN-SBR Fwd 5′ctgctcgtagaagccgagag3′; JUN-SBR Rvs 5′gcgcccactataaaaactgc3′; JUN-TSS Fwd 5′gctggctgtgtctgtctgtc3′; JUN-TSS Rvs 5′gggtgacatcatgggctatt3′. A standard curve was calculated from 8 different dilutions of input DNA, which was used to normalize the qPCR data from the IPs. Beads alone incubated with extract in the absence of antibody were used as a control for non-specific genomic DNA binding.

### Statistical analysis

In all statistical analysis shown in the figures, data represent mean ± SEM. Data was analyzed by ANOVA analysis. In all cases, we used Prism’s Graph Pad software for data calculation and representation. At the figure legends, the asterisks denote statistically significant differences between the treatments (*p < 0.05, **p < 0.005 and ***p < 0.001, ****p < 0.0001).

## Results

### Sirt1 interacts with Smad2

In order to look for new players involved in the regulation of the transcriptional responses to TGFβ, we used the Cyto Trap Two-Hybrid System. This Cyto Trap version allows working with transcriptional factors such as Smad2, as bait/target interactions occurs in the cytosol and not on a promoter. Human Smad2 was cloned along with Smad4 and the positive interaction test of both factors was considered as a proof for the reliability of the system. A library from human lung epithelial cells was then used to perform a screening for Smad2-interacting proteins. We found 300 different protein-representing clones positively interacting with Smad2, which corresponded to 110 proteins (data not shown), including human Sirt1. Sequence analysis of the positive Sirt1 clone showed it corresponded to a full length open reading frame and revealed lack of any mutations when compared to the human genome database.

**Fig. 1:**
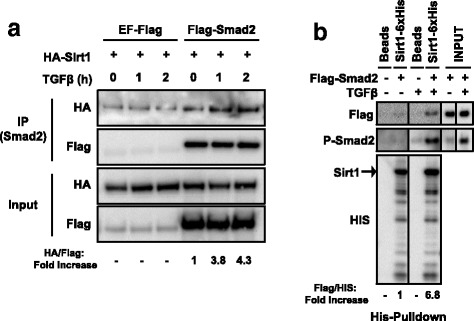
Sirt1 interacts with resting and active Smad2 in vitro and in vivo. **a** Hep3B cells transfected with HA-Sirt1 and either Flag-Smad2 or EF-Flag were treated with TSA for 8 h followed by TGFβ for the indicated times. Smad2 was purified by immunoprecipitation and Sirt1 or Smad2 were detected by Western Blot. Fold increase (lane 4–6 over lane 4) of HA/Flag levels are indicated at the bottom. Weak Sirt1 binding to control samples were taken into account for calculations. **b** HEK293T cells transfected with Flag-Smad2 were treated overnight with SB-431542, then washed out and treated with TGFβ for 1 h. Flag-Smad2 was purified with a flag peptide competitor and used for His-Pull-down assay. Flag-Smad2, PSmad2 and Sirt1-6xHis were detected by Western Blot. Fold increase (lane 2 and 4 over lane 2) of Flag/HIS levels are indicated at the bottom. The vertical lines across the blot indicate that two distant parts of the very same blot were put together. Lanes are considered left to right. All the experiments of this figure were repeated at least three times. Representative results are shown. IP: Immunoprecipitation

To check whether Sirt1 binds to Smad2 in vivo, HA-Sirt1 and Flag-Smad2 were overexpressed in Hep3B cells. Following treatment with TSA, which broadly inhibits protein deacetylation [11], cells were stimulated with TGFβ and Smad2 was purified by immunoprecipitation. Although Sirt1 exhibited certain tendency to stick (see Materials and Methods), immuno-blots revealed that Sirt1 and Smad2 specifically interacted in HEP3B cells in vivo*,* and that the interaction seemed to be enhanced by TGFβ stimulation (Fig. 1a). Moreover, by using an in vitro protein pull-down applying His tagged Sirt1 beads and Flag tagged Smad2 purified from TGFβ-stimulated HEK293T cells, we could observe that the interaction Smad2/Sirt1 was clearly enhanced when TGFß activated, i.e. phosphorylated Smad2, was used (Fig. 1b).

Altogether, these results imply that Sirt1 and Smad2 interact both in vitro and in vivo. Moreover, our data suggest that this interaction can happen in the absence of TGFβ stimulation, although it is enhanced by TGFβ ligand.

### Interaction with Sirt1 is modulated by Smad2 acetylation status

**Fig. 2:**
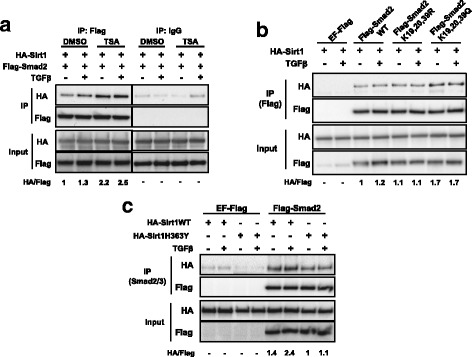
The interaction between Sirt1 and Smad2 is enhanced by Smad2 acetylation. **a** HEK293T cells transfected with the indicated plasmids were treated with SB-431542 and TSA or DMSO during 14 h, then washed out and treated with TGFβ for 1 h. Flag-Smad2 was immunoprecipitated and Smad2 and Sirt1 were detected by Western Blot. HA/Flag fold increase is indicated (lane 1–4 over lane 1). Weak Sirt1 binding to control samples were taken into account for calculations. The vertical lines across the blot indicate that two distant parts of the very same blot were put together. **b** HEK293T cells transfected with the indicated plasmids were treated with SB-431542 overnight, then washed out and treated with for 1 h. Flag proteins were immunoprecipitated and Sirt1 or Smad2WT and mutants were detected by Western Blot. HA/Flag fold increase is indicated (lane 3–8 over lane 3). **c** Hep3B cells transfected with the indicated plasmids were serum starved overnight and treated with TSA for 8 h followed by TGFβ for 1 h. Smad2/3 was purified by immunoprecipitation. Sirt1WT, Sirt1H363Y and Smad2 were detected by Western Blot. HA/Flag fold increase is indicated (lane 5–8 over lane 7). Weak Sirt1 binding to control samples were taken into account for calculations. All lanes are numbered from left to right. IP: immunoprecipitation, WT: wild type. All the experiments of this figure were repeated at least three times. Representative results are shown

Upon TGFβ stimulation, Smad2 and Smad3 are activated by phosphorylation. Additionally, it has been reported that TGFβ induces the acetylation of Smad2 at lysine residues 19, 20 and 39 [9, 11]. Indeed, immunoprecipitation assays of cell extracts from Hep3B cells treated with TSA showed an increase of Smad2 acetylation up to 2 h after TGFβ stimulation (Additional file 1: Figure S1a). In order to clarify whether Smad2 acetylation status had an impact on the interaction with Sirt1, we co-transfected HEK293T cells with Flag-Smad2 and HA-Sirt1 and performed immunoprecipitation studies after treatment with or without TGFβ and TSA. Flag immunoprecipitation of these cell extracts revealed that inhibition of types I and II deacetylases significantly enhanced the interaction between Sirt1 and Smad2, while TGFβ treatment showed some influence on the interaction (Fig. 2a) (Weak Sirt1 binding to control samples were taken into account, see above). To further study the involvement of the acetylation of Smad2 lysine residues 19, 20 and 39 in the Smad2/Sirt1 interaction, we generated two Smad2 mutants which were co-transfected along with HA-Sirt1 into HEK293T cells: a first one which cannot be acetylated (Smad2^K19R, K20R, K39R^); and a second one which is functionally similar to the fully acetylated form (Smad2^K19Q, K20Q, K39Q^) (Additional file 1: Figure S1b) [11]. HA-Sirt1 immunoprecipitation of Flag proteins showed that Sirt1/Smad2^K19Q, K20Q, K39Q^ interaction was stronger as compared to acetylation-devoid and native Smad2 forms (Fig. 2b). To test whether Smad2/Sirt1 interaction could be conditioned by Sirt1 catalytic activity, we engineered the catalytically inactive mutant version Sirt1^H363Y^ (Additional file 1: Figure S1c) [34]. Extracts obtained from Hep3B cells co-transfected with Flag-Smad2 and either HA-Sirt1 or HA-Sirt1^H363Y^ were immunoprecipitated for Flag (Smad2). Interestingly, both native and mutant Sirt1 proteins exhibited similar level of interaction with Smad2, although slightly higher interaction was observed for wild type Sirt1 in the case of TGFβ-stimulated cell extracts (Fig. 2c).

Jointly, these data suggest that, although Sirt1/Smad2 interaction may occur in the absence of stimulation, it is favored by Smad2 acetylation. Moreover, our data support that this interaction might be partially dependent on Sirt1 catalytic activity.

### The interaction of both proteins occurs via Smad2 globular domains and Sirt1 N-terminal segment

**Fig. 3:**
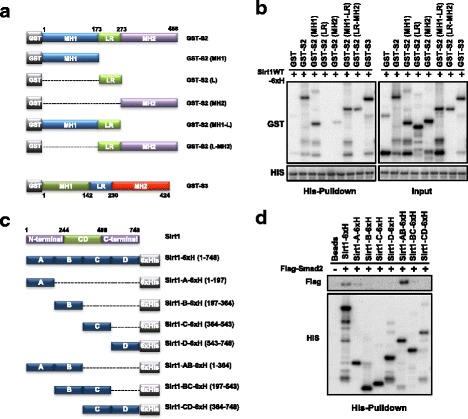
The interaction occurring between Sirt1 and Smad2 is domain-specific. **a** Schematic representation of full length GST-Smad2 and GST-Smad3 and different GST-Smad2 fragments. **b** A His-Pull-down assay was performed to determine interaction between Sirt1WT-6xHis and full-length GST-Smad2 or GST-Smad3, or GST-Smad2 fragments. Different constructs were detected by Western Blot. **c** Schematic representation of full length Sirt1WT-6xHis and different Sirt1-6xHis fragments. “CD”: catalytic domain. **d** His-Pull-down assay to determine interaction between Flag-Smad2 and Sirt1-6xHis and different Sirt1-6xHis fragments. Flag-Smad2 was expressed in HEK293T cells and purified with a flag peptide. Different constructs were detected by Western Blot. All the experiments of this figure were repeated at least three times. Representative results are shown

The previous results prompted us to study the Smad2 and Sirt1 proteins domains that mediate the interaction. As preliminary observations were made on Smad2 Cyto trap, we decided to engineer different fusion-proteins composed of GST and Smad2 domains (Fig. 3a). His-pull-down showed that full length GST-Smad2 and GST-Smad3 were able to directly bind to His-tagged Sirt1, while GST protein alone did not interact with Sirt1 (Fig. 3b). Interestingly, when portions of Smad2 were used, we found that MH1 domain interaction with Sirt1 had similar affinity than full length Smad2, in contrast to MH2 domain that showed weak affinity. Strikingly, although Linker domain alone did not interact, the GST-Linker-MH2 fusion protein showed similar affinity for Sirt1, as the MH1 alone or the Linker-MH1 domains (Fig. 3b). The catalytically inactive mutant Sirt1^H363Y^ revealed a similar interaction pattern with full length Smads and Smad2 fragments to that observed with wild type Sirt1 (data not shown). To investigate which domains of Sirt1 might be involved in the interaction with Smad2, pull-down assays, using different fragments of Sirt1 fused to His tag, were also performed to identify interaction domains with Flag-Smad2 (Fig. 3c). Interestingly, the strongest interaction was reported for the AB fragment, while interaction with A or B fragments separately were very weak or undetectable (Fig. 3d).

Altogether, these results strongly suggest that Smad2-Sirt1 interaction is direct and occurs via motifs located in the globular MH1 and MH2 domains of Smad2, and in the N-terminal, residues 1 to 364, of Sirt1.

### Sirt1 and Smad2 colocalize in response to TGFβ

**Fig. 4:**
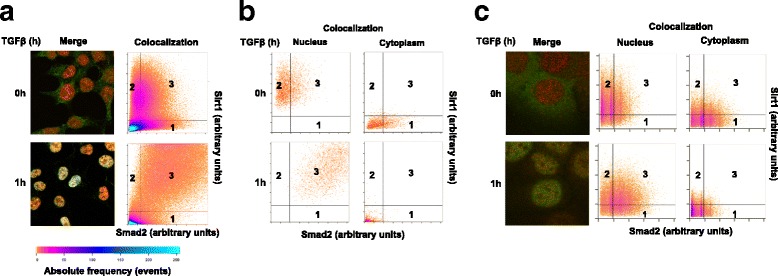
Sirt1 and Smad2 co-localize in the nucleus after TGFβ treatment. **a** HEK293T cells were treated with SB-431542 overnight, the washed out and treated with TGFβ for the indicated times. Cells were immuno-labeled for Smad2 (green) and Sirt1 (red). Plots show Sirt1-Smad2 colocalization (pixel fluorescence intensity in each channel) for whole cells. **b** Plots show Sirt1-Smad2 colocalization for single HEK293T cell’s nucleus and cytoplasm separately. X-axis: Smad2-bound fluorophore intensity. Y-axis: Sirt1-bound fluorophore intensity. **c** HaCaT-GFP-Smad2 cells were treated with TGFβ for the indicated times, then immuno-labeled for Sirt1. Plots show Sirt1-Smad2 colocalization for single cell’s nucleus and cytoplasm. Detection areas: (1) green fluorescence; (2) red fluorescence; (3) colocalization. Color bar: absolute frequency range. All the experiments in this figure were repeated at least three times. Representative results are shown

Nuclear localization of Smad2 and Smad3 has been related to their role in TGFβ-dependent transcriptional activation [32, 41]. As an enhancement of Sirt1/Smad2 interaction was observed upon Smad2 activation and subsequent acetylation, we decided to study the subcellular localization of Smad2 and Sirt1 in response to TGFβ stimulation. Sirt1 typically has a nuclear localization [42], that can vary during embryo development or depending on either the tissue type or cell differentiation [43]. Immunostaining of HEK293T cells exhibited a predominantly nuclear staining of Sirt1 with a weak cytosolic localization, and the usual Smad2 cytosolic and nuclear localization [32]. Upon TGFβ treatment, Sirt1 labeling appeared to be slightly more abundant in the nucleus while Smad2, as expected, turned completely nuclear (Additional file 2: Figure S2a). Interestingly, confocal microscope image co-localization analysis of whole cell-fields revealed, in cells treated with TGFβ, an increase of pixels with high fluorescence intensity for both proteins, in comparison with untreated cells which exhibited weaker co-localization (Fig. 4a). In this fashion, when we analyzed separately the nuclear region of single cells, co-localization plots depict an increase of high intensity pixels for both Sirt1 and Smad2 into the nucleus of TGFβ treated cells, in contrast to untreated cells in which very little nuclear Smad2 immunostaining, and thus poor colocalization with Sirt1, is observed (Fig. 4b). On the contrary, when analyzing the cytosolic region, intense Smad2 immuno-labelling quickly vanished upon TGFβ treatment, whereas the poor Sirt1 immuno-labelling contribution moved away as well (Fig. 4b). In an effort to further corroborate those observations, we applied a similar strategy using HaCaT cells stably expressing GFP-Smad2 [32]. TGFβ-stimulated HaCaT-GFP-Smad2 cells show intense nuclear staining both for GFP-Smad2 and Sirt1 (Additional file 2: Figure S2b). Also in this case, the colocalization analysis reveals an increase of high fluorescence intensity pixels for both markers in the nucleus, along with an obvious cytosolic decrease after TGFβ treatment (Fig. 4c). It is worth noting that in our hands, HEK293T and HaCaT cells did not show changes in Sirt1 expression in response to TGFβ stimulation (Data not shown).

These data indicate that, in cultured cells, Sirt1 and Smad2 may share very close, roughly the same, topological localizations in the nucleus of cells treated with TGFβ.

### Sirt1 interacts with Smad complexes on DNA in vitro

**Fig. 5:**
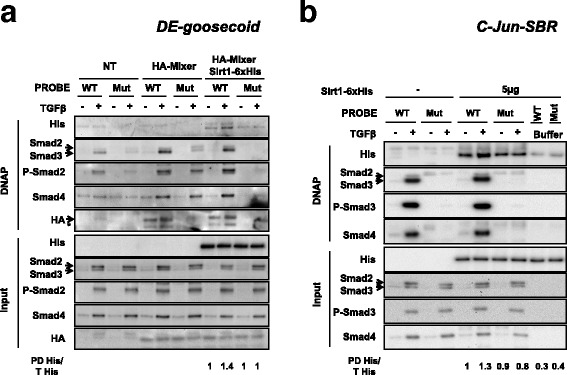
Sirt1 detection on different regulatory elements is increased after TGFβ treatment. Nuclear extracts from HaCaT cells treated or not with TGFβ for 1 h were used for DNA pull-down assays. **a** Extracts were supplemented with Sirt1-6xHis and HA-Mixer-transfected HEK293T nuclear extracts. Pull-down was performed with specific oligonucleotides for regulatory sequence *DE-goosecoid* and mutant oligonucleotides for Smad2/Smad4 binding. Antibodies used for Western Blot detection are indicated. Pull down Sirt1-6xHis (PD His) and total Sirt1-6xHis were quantified. PD His/T His is indicated (lane 9–12 over lane 9). **b** Extracts were supplemented with Sirt1-6xHis. Pull-down was performed with specific oligonucleotides for regulatory sequence *c-jun*-*SBR* and mutant oligonucleotides for Smad3/Smad4 binding. The indicated antibodies were used for Western Blot detection. Pull down Sirt1-6xHis (PD His) and total Sirt1-6xHis were quantified. PD His/T His is indicated (lane 5–10 over lane 5). All the experiments of this figure were repeated at least three times. Representative results are shown. WT: Wild Type oligonucleotide. Mut: mutant oligonucleotide. Buffer: no-input control. DNAP: DNA pull-down. NT: not transfected. (*): unspecific band

To clarify whether Sirt1/Smad2 interaction could take place during Smad2-driven regulation of TGFβ-dependent promoters, we studied Sirt1 presence on the DE element of the *goosecoid* promoter from *Xenopus laevis*. Within this system, Smad2 binding to the DE element is conditioned by the presence of the *Xenopus *transcription factor Mixer [33, 44, 45]. Nuclear extracts from HaCaT cells stimulated with TGFβ were supplemented with HA-Mixer, purified from transfected HEK293T cells, and/or Sirt1-6xHis, purified from bacteria, then used for DNA-pull-down assays for the DE elements. In the absence of Mixer, recovery of either activated-Smad2 or Smad4 was poor with the wild type DE probe, and negligible when DE mutant probe was used, despite TGFβ stimulation (Fig. 5a). As expected, when Mixer was present, recovery of both activated-Smad2 or Smad4 was patently enhanced from TGFβ-stimulated nuclear extracts. Interestingly, the presence of Smad3 was also revealed in these complexes (Fig. 5a). Sirt1 recovery, within the Smad2-Smad4-Mixer setup, was very poor in the absence of TGFβ or with the mutant DE probe. However, when TGFβ was present, Sirt1 recovery from the wild type DE probe clearly increased (Fig. 5a).

To further complement the aforementioned observations, HaCaT nuclear extracts, supplemented with purified His tagged Sirt1, were used to examine the presence of Sirt1 on the Smad Binding Region (SBR) of the *c-jun* promoter, by DNA-pull-down assays. Immunoblots showed that Sirt1 presence on DNA can be detected independently of probes and of TGFβ stimulation. However, Sirt1 presence on SBR was clearly enhanced with nuclear extracts from TGFβ-stimulated cells, i.e. in the presence of activated Smad2 (Fig. 5b). Strikingly, this only occurs when wild type probe is used, which also promotes recruitment of phosphorylated Smad3, Smad4, along some levels of Smad2. Interestingly, the presence of Sirt1 neither interfered nor improved the Smads recruitment to DNA. Strikingly, Sirt1 recovery was highly impaired in the absence of nuclear extract proteins (Fig. 5b).

Altogether, our DNA-pull-down results indicate that Sirt1 interaction with DNA is favored by the presence of nuclear proteins, and further increased specifically on Smad-driven promoters upon TGFβ stimulation. This recruitment process would not compromise the access of Smad proteins to their target sequences.

### Sirt1 is recruited to TGFβ regulated genes in vivo

**Fig. 6:**
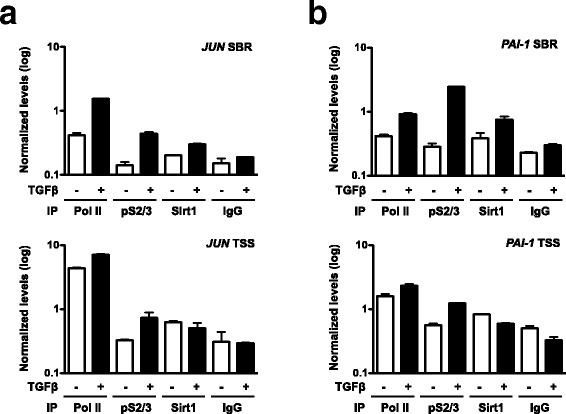
Sirt1 accumulates on Smad regulating elements in response to TGFβ treatment in HaCaT cells. Cells were kept overnight in OPTIMEM culture medium and stimulated with TGFβ for 1.5 h. **a** and **b**. ChIP analysis of the SBR and TSS regions of the *JUN* gene (**a**) and the SBR and TSS regions of the *PAI-1* (**b**) gene using antibodies against RNA PolII, phosphorylated-Smad2/3, Sirt1 and control IgG. Shown data are means and standard deviations of qPCRs performed in triplicate in a representative experiment

In order to test if the observed Sirt1 access onto Smad-regulated promoters indeed occurs in culture, we performed chromatin immuno-precipitation (ChIP) assays from HaCaT cells stimulated with TGFβ for 90 min. Real time qPCR was performed on ChIP eluates to reveal the presence of regulatory regions of two TGFβ-target genes, *JUN* and *PAI-*1. In these genes, the regions corresponding to SBR and to the transcription start site (TSS) were studied. Recovery from anti-phosphorylated-Smad2/3 and anti-RNA polymerase II immunoprecipitations were used as optimal TGFβ stimulation readouts. As Sirt1 presented with DNA binding affinity (see Fig. 5), also its main function relates to histones metabolism, it was not surprising to find detectable recovery levels of both SBR and TSS regions from anti-Sirt1 ChIP reactions of unstimulated cells. However, upon TGFβ stimulation, recovery of SBR region of JUN from anti-Sirt1 reactions was increased, while that of TSS remained stable (Fig. 6a). Similarly, for *PAI-1* gene regulatory regions (Fig. 6b), recovery of the SBR from anti-Sirt1 ChIP reactions showed an increase, which again was not observed for the TSS region. As it can be observed, although recovery from anti-phosphorylated-Smad2/3 ChIP reactions was generally increased in TGFβ stimulated samples, this trend was matched by anti-Sirt1 reactions for the SBR regions, in which the enhancement of phosphorylated-Smad2/3 recovery was in fact more apparent.

Conjointly, these results support the notion that Sirt1 can be actively recruited along with Smads onto TGFβ-regulated gene promoter regions.

### Sirt1 activity can modulate the transcription of TGFβ-dependent genes

**Fig. 7:**
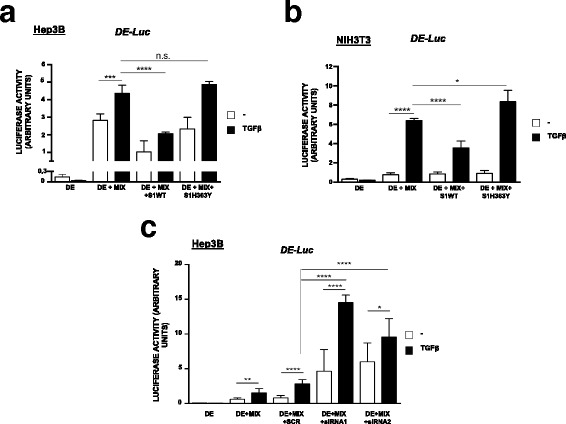
Sirt1 specifically repress TGFβ dependent transcriptional activity of DE-Luc reporter gene. **a** and **b**. Hep3B (**a**) and NIH3T3 (**b**) cells were transfected with the indicated plasmids. After 48 h cells were treated with TGFβ for 6 h and measured for luciferase activity. **c** Hep3B cells were additionally transfected with Sirt1 siRNA plasmids. After 48 h cells were treated with TGFβ for 6 h and measured for luciferase activity. “MIX”: Mixer transcription factor. “SCR”: Scramble sequence. Luciferase activity represents firefly luciferase activity normalized by Renilla activity. Asterisks denote significant differences according to ANOVA statistical analysis: *p < 0.05; ***p < 0.005 ***p < 0.001; ****p < 0.0001

We next decided to study whether the observed Sirt1/Smad-complexes interaction may control the transcription of TGFβ target genes. For that purpose, we used a luciferase assay to study potential effects on two Smad2 TGFβ dependent *Xenopus laevis* genomic regions: the *goosecoid* promoter (*DE-Luc*) and the *Mix.2* promoter (*ARE-Luc*). Smad2/Smad4 complexes require additional transcription factors to activate transcriptional activity of these reporter genes, Mixer in the case of *DE-Luc*, and FoxH1 for *ARE-Luc* [33]. In Hep3B cells transfected with the DE-Luc reporter, luciferase assays revealed how Sirt1 overexpression partially repressed TGFβ-dependent transcription induction. On the other hand, it also affected, to some extent, the activity detected in resting cells. Interestingly, overexpression of the catalytically inactive mutant Sirt1H363Y did not cause any significant luciferase activity alteration within this setup (Fig. 7a). Furthermore, when we applied the same approach on NIH3T3 cells similar results were obtained, as Sirt1 overexpression effectively reduced TGFβ dependent luciferase activity. In this case, overexpression of Sirt1H363Y increased significantly TGFβ dependent transcriptional activity (Fig. 7b). Additionally, when we studied if Sirt1 could affect the activity derived from the *ARE-Luc* expression system, again, luciferase assays reported reduced activity for NIH3T3 cells overexpressing Sirt1, while a slight yet not significant increase of activity was observed for the case of Sirt1H363Y (Additional file 3: Figure S3a).

To further explore the contribution of endogenous Sirt1 on TGFβ-dependent gene transcription, we performed a Sirt1 interference study in combination with the luciferase strategy. Two different siRNA for Sirt1 were designed, cloned into pTER-GPF plasmid and transfected into Hep3B cells. The interference efficiency of both siRNA was assessed by means of confocal microscopy (Additional file 3: Figure S3b) and western blot analysis of transfected cells, with average reduction of Sirt1 expression levels of 82% and 79% for siRNA1 and siRNA2, respectively (Additional file 3: Figure S3c). Assays performed on cells carrying either siRNA1 or siRNA2 constructs together with the DE-Luc system clearly show how, upon TGFβ stimulation, luciferase activity was increased far beyond the levels achieved by scrambled-siRNA or non-TGFβ-stimulated cells (Fig. 7c). It is worth noting that basal luciferase activity was also increased by the Sirt1 siRNA (Fig. 7c).

Summing up, luciferase assays results support the notion that Sirt1 has access to Smad-driven promoters and it is recruited to these regions upon TGFβ stimulation. Moreover, our results suggest that its deacetylase activity may provide an inhibitory input on the regulation of TGFβ dependent gene transcription.

## Discussion

In this work, by using a two-hybrid chimera interacting system, we provided first evidence of a direct interaction involving Sirt1 and Smad2. Subsequent analyses confirmed the interaction and allowed to characterize the relevance of the Smad2 acetylation status for the molecular interplay. Moreover, we determined that the interaction involves Sirt1 recruitment into functional complexes on DNA, both in vivo and in vitro, thereby affecting transcriptional output and supporting previous observations involving Smad factors and Sirt1. Our work has implications in the understanding of Smad-mediated gene expression regulation.

In the past, conventional Two-Hybrid Systems had been successfully used to identify proteins interacting with Smads [46–50]. However, these systems, which relied on the reconstitution of a functional Gal4 transcription activator, pose some limitations to its use with proteins that are capable to activate transcription on their own, i.e. to transcription factors [51, 52]. In our case, we used the Cyto Trap Two-Hybrid System, which rely on interactions occurring in the cytosol and thus overcome such limitations and provide greater sensitivity. Implementing an expression library from human lung epithelial cells within this system allowed performing a screen for Smad2-interacting proteins and identifying a set of 110 different proteins that positively interacted with Smad2. From the whole set of identified sequences, computational analysis helped in (i) detecting proteins previously known to interact with Smad2, as MAN1 or CKIe [53, 54]; (ii) noticing proteins which have been described to be connected to TGFβ signaling, as ICAM1 or HEF1 [55, 56]; and (iii) identifying new potential players interacting with Smad2. Among the protein set, which interactions with the TGFβ signaling pathway and Smad2 was not previously reported, a complete ORF clone for Human Sirt1 was isolated and identified. Strikingly, while different reports indicated in the past the ability of Sirt1 to interact with Smad3 and possibly Smad4 [22, 26], its interaction with Smad2 had remained elusive so far. In cultured Hep3B cells, we demonstrated that HA-Sirt1 and Flag-Smad2-tagged products effectively interacted. Altogether, the small number of positive clones obtained within the cytosolic Two-Hybrid system, along with the confirmation of the Smad2/Sirt1 interaction, supports the suitability and convenience of the approach used. Moreover, taking in account the number of previous established interactions revealed in the analysis, this confirms the reliability of this system for the characterization of subtle protein interactions occurring natively in the nucleus, as is the case with Smad2 and Sirt1.

Mammalian sirtuins are orthologous of the silent information regulator-2 gene, which in yeast was described to extend life span. They comprise a family of proteins (Sirt1–7), usually described as NAD+ dependent type III histone deacetylases (HDACs), which perform a gatekeeping role in the configuration of the cell transcriptome. However, in these last years, additional competences had been described for several sirtuins members [15], which is in line with similar observations involving type I and type II HDACs. Precisely Sirt1, on top of its default role as histone deacetylase, has also emerged as an important modulator of the activities of relevant non-histone nuclear proteins involved in transcriptional regulation, among which p53, FOXO, E2F1 or NF-kB [16–18]. Consequently, this myriad of targets confers great influence of Sirt1 activities in regulating a significant portion of the proteome, as evidenced by its relation to an array of comprehensive biological processes and associated pathologies, including development, cellular senescence and aging or inflammation, with an important role in the modulation of energy metabolism [19–21]. The family of Smad signal transducers comprises eight members in humans, five of which (1, 2, 3, 5, 8), are called receptor-regulated Smads (R-Smads) as they are phosphorylated in their C-terminal end in response to TGFβ (Smad2 and 3) or BMP (Smad1, 5 and 8) ligand binding to their receptors. Phosphorylated R-Smads then integrate in a heterotrimer with Smad4, the co-Smad, which contributes in the recruitment of additional factors required for optimal TGFβ transcriptional regulation, through interaction with complementary Smad Binding Elements (SBEs) at the chromatin or directly binding to specific transcription factors on chromatin [3, 57, 58]. In addition, TGFβ signaling is negatively regulated via the I-Smad, Smad6 and Smad7. Smad7 plays a key role, as it functions as an inhibitory factor blocking Smad2 access to the active TGFβ receptor.

TGFβ signaling is subjected to fine modulation involving posttranslational modifications of the many pathway molecular transducers, including acetylation and deacetylation. Interestingly, deacetylation of Smad7 has been shown to target the factor for ubiquitination and later proteasome degradation [12]. Smad2 is also known to be acetylated at the lysine residues 19, 20 and 39 [9, 11], in response to TGFβ stimulation [7, 10, 11]. This process is crucial for optimal and sustained TGFβ transcriptional regulation, since acetylation by CBP/p300 and PCAF retains activated R-Smads in the nucleus, by slowing down their shuttling back to the cytoplasm [9–11]. Interestingly, Sirt1 is capable to bind and deacetylate phosphorylated Smad3, thereby decreasing Smad3 transcriptional activity [22]; similar dynamics had been also suggested in the case of Smad4 [11, 26]. Indeed, in our hands, GST-Smad3 was able to interact with Sirt1WT-6xHis. More importantly, the Smad2-Sirt1 interaction was sustained when cells were stimulated with TGFβ. This phenomenon, which was further confirmed in vitro by protein pull-down assays from TGFβ stimulated HEK293T cells, would be coherent with the ability of TGFβ to promote acetylation of R-Smads.

Although not characterized yet, type-I and type-II deacetylases are known to take part in modulating the dynamics of Smad2 acetylation. Consequently, treatment with TSA, a specific inhibitor of type-I and -II deacetylases, is known to favor the acetylation of Smad2 [11]. This has particular relevance in the case of HEK293T cells, which express low p300 levels [11]. Interestingly, while Sirt1 catalytic activity is not affected by TSA treatment [59, 60], our immunoprecipitation assays from TSA treated samples distinctly showed enhanced Sirt1-Smad2 interaction. Whereas this result would confirm the ability of both proteins to directly interact, it could also indicate that the interaction would be dependent on Smad2 acetylation status, thus envisaging a role for Sirt1 in deacetylating Smad2. In that sense, Smad7, which acetylation status is increased by TSA, is known to be deacetylated by complexes integrating Type-I and Type-II deacetylases along with Sirt1 [13, 14]. It is important to mention that p53 has been shown to be subjected to a similar modulation mechanism with the contribution of Sirt1 [34, 61, 62]. Surprisingly, our data suggest that Sirt1, although better binding Smad2 when it is catalytically active, would not play a main part in deacetylating Smad2, as over-expression of wild-type Sirt1 did not alter significantly acetylated-Smad2 levels (data not shown). However, acetylation status of Smad2 may be important to promote the Smad2/Sirt1 interaction. Indeed, acetylation mimicking Smad2 mutants interacted strongly with Sirt1 in HEK293T cells, as compared to native Smad2. In any case, the fact that the Sirt1-Smad2 interaction could be similarly detected independently of TGFβ stimulation-dependent acetylation, as revealed by using both the native Smad2 protein or the acetylation deficient Smad2 mutant, confirms the existence of a strong interplay between both factors and TGFß signalling. Notably, this circumstance could enable for augmented transcriptional modulation, since Sirt1 recruitment depending on Smad2 activation and nuclear acetylation could potentially affect the acetylation status and the activities of different transcription factors integrating the Smad2 driven transcriptomic complexes.

Protein subcellular localization is important to understand protein function. While Smads nucleocytoplasmic shuttling is crucial for proper transduction of TGFβ signaling [63], Sirt1 typically shows a nuclear localization [42] which could vary in different situations [43]. In our case, the interaction between Sirt1-Smad2 detected using molecular techniques was also evidenced by means of immunofluorescence labeling. Confocal image co-localization analysis allowed us to further characterize this interaction based in measuring individual pixels on merged fluorescence fields. Both fluorophores are represented and related using fluorescence intensity levels. Surprisingly, rapidly after TGFβ stimulation, numerous pixels appeared concentrating into the nucleus, showing high intensity for both markers. This phenomenon was similarly observed in the two cell lines used. This concomitance could be deemed to Sirt1 being recruited to active genes in response to histone acetylation changes. However, previous literature indicating Sirt1 deacetylase activities over Smads [11, 26] also suggested the possibility that Sirt1 specifically integrated complexes at TGFβ-regulated promoters. In that sense, DNA-pull-down assays performed using the validated TGFβ-dependent promoter constructs *DE-goosecoid* and *c-jun-SBR*, allowed us to corroborate recruitment of Sirt1 onto promoters along with Smads in response to TGFβ in vitro, thus in the absence of histone/chromatin. Although we observed the binding of Sirt1 to DNA in the absence of TGFβ signaling and independently of Smad binding elements, a clearer and stronger signal for Sirt1 was detected upon activation of Smads. It has been suggested that nuclear proteins may be involved in a scanning process to locate specific binding sites [32, 64]. Despite Sirt1 lacks a DNA binding domain, it relates to it by interacting with different DNA-binding nuclear proteins [65, 66]. This would explain the detection of Sirt1 on DNA in the absence of DNA-bound Smads but in the existence of such nuclear proteins. Nevertheless, the presence of TGFß-activated Smads robustly and specifically enhanced the binding of Sirt1 to *DE-goosecoid* and *c-jun-SBR.*

In the same line, the development of ChIP assays for well-established TGFβ-dependent promoters further supported recruitment of Sirt1 in vivo*.* As Smad2-Smad4 interaction only occurs upon TGFβ stimulation, R-Smads activation and translocation to the nucleus [57], these results would support the notion that Sirt1 joins active Smads-borne transcription complexes. Thus, although these experimental setups cannot exclude the natural presence of Sirt1 related to histone acetylation modulation in response to transcription activation, it is our understanding that these observations strongly point to a specific role for Sirt1 in modulating TGFβ-dependent transcription regulation, as the presence of factors on DNA-bound protein complexes is usually regarded as denoting a transcriptomic functional contribution [67]. Moreover, these findings strongly suggest that spatial co-localization observed for both Sirt1 and Smad2 in the case of TGFβ stimulated cells, would be the consequence of their concomitant recruitment onto the vicinity of SBEs in vivo.

All mammalian Sirtuins (Sirt 1–7) are characterized by a conserved 275 amino acid catalytic core domain [16]. This core is flanked by N-terminal and C-terminal sequences of variable length and composition, which influence to different extents the enzymatic activity [68]. However, relatively little is known about the regulatory mechanisms modulating Sirt1 activity. On the other hand, Smads structure and physiology are better understood. Smad4 and R-Smads are composed of two conserved globular domains, named MH1 and MH2, held together by a poorly conserved Linker region [3]. On the N-terminal part the MH1domain, while able to interact with some factors like c-JUN or SP1, is mainly involved in DNA binding, directly as in the case of Smad3, or through transcription factor interaction for Smad2 [69]. At the C-terminal end, the MH2 domain usually mediates protein-protein interaction with effector proteins, including the TGFβ receptors and different cofactors and chromatin modifiers, including histone acetylases and deacetylases. Importantly, the central linker region incorporates additional posttranslational modification sites [3, 8]. Intriguingly, although it is acknowledged that mutations in the MH2 domain are linked to dysfunctional TGFβ signaling, epithelial-mesenchymal transition (EMT) and cancer, no common recognition motifs have been determined for interactions with this domain [28, 70]. Our observations indicate that despite the Smad2 MH2 shows some affinity for Sirt1, the combination of the linker with the MH2 domain displayed greater interaction. Interestingly, for both Smad3 and Smad4, it has been described that close to the MH2, the linker has a proline-rich area, the Smad activation domain (SAD), responsible for interactions with p300 [71], and that participates in R-Smads acetylation. Intriguingly, we also found that Sirt1 strongly interact with the MH1 domain of Smad2. Worth reminding, functional acetylation of residues at positions 19, 20 and 39 in this domain resulted in increased interaction with Sirt1. Notably, Blast sequence comparisons (data not shown) between Smad2 and Smad7 reveal considerable homology in this area, a feature that is also shared by Smad3. In Smad7, the N-terminal domain mediates interaction with p300 and Sirt1, which acetylate and deacetylate lysine residues in the same region [14].

Regarding Sirt1, we found that the interaction with Smad2 only involved the non-catalytic N-terminal segment. The N-terminal domain has been recently described to be responsible of mediating interaction with the NF-kB subunit, p65, and thus to promote its deacetylation [72], while Sirt1 C-terminal segment has been shown to mediate interaction with inhibitors [73]. Moreover, SKI-interacting-protein (SKIP), an essential co-regulator known to potentiate the activity of different transcription factors including R-Smads, has been demonstrated to interact within the same Sirt1 N-terminal region [74, 75]. Thus, all those evidences strongly support the notion that relevant interactions occur through the domains of Sirt1 and Smad2, identified in our study. As mentioned earlier, literature reports that whereas Smad3 directly binds to target DNA sequences, Smad2 interaction with DNA apparently requires the concurrence of additional factors [58]. Interestingly, this opens the possibility for a role of Smad2 in recruiting Sirt1 as a common regulator of associated transcription factors physiology. In that context, it should be noted that in EMT, usually considered as a hallmark of TGFβ deregulation [28], Sirt1 has been reported to play both supportive and suppressive roles [25–27]. In any case, considering that our data did not allow to fully establish the root for Sirt1-Smad2 interaction but confirmed Sirt1 recruitment on Smad/DNA complexes, we further studied the significance of the interaction. For that purpose, we designed experimental setups relying on the specific Smad2-dependent expression constructs DE-Luc and ARE-Luc [33]. Strikingly, results in over-expression assays revealed that increased native Sirt1 activity decreased reporter gene outputs. In contrast, results in interference assays coherently showed that reduced Sirt1 activity resulted in increased reporter gene activity. This increase was not only detected when cells were stimulated with TGFβ but also at the resting state, suggesting that a Sirt1-mediated basal promoter repressing activity may be at play. This would be in line with the relatively high amount of Sirt1 found at the ChIP assays. The sum of these data, while being in line with previously characterized interactions of Sirt1 with Smad3, and seemingly Smad4 [11, 22, 26], also verified the functional relevance of this interaction specifically regarding transcriptional complexes involving Smad2.

The nature of TGFβ signaling is complex and diverse. A better understanding of the parallel mechanisms capable of modulating its final output should therefore help to decipher the complex regulation of this signaling and its diversity of outcomes. As the increasing body of literature dealing with HDACs interactions with non-histone factors points, this interplay provides an adequate framework to further characterize developmental processes. In this respect, and due to the pleiotropic nature of both TGFβ signaling as well as of Sirt1 activities, the possibility of Smad2 acting as a hub for Sirt1 action in the tight context of transcriptomic complexes, draws a special line of research that we intend to work through in the future, potentially opening opportunities for the development of innovative strategies to address diverse biological processes and pathologies including cancer.

## Conclusions

- A yeast two-hybrid protein–protein interaction assay identified Sirt1 as novel interactor for Smad2.
- This interaction was confirmed to involve both resting and active Smad2.
- The interaction between Sirt1 and Smad2 was enhanced by Smad2 acetylation, involving specific domains.
- Sirt1 presence on TGFβ dependent regulatory elements was found increased after TGFβ treatment using different techniques.
- Sirt1 overexpression resulted in repression of a TGFβ dependent reporter gene assay.

## Additional files

Additional file 1: Figure S1. TGFβ induces the acetylation of Smad2. (a) Hep3B cells were serum starved overnight, treated with TSA for 8 h followed by TGFβ for the indicated times. Smad2 was purified by immunoprecipitation, then acetyl-Lys,Smad2, Smad4 and PSmad2 were detected by Western Blot. Ack/Smad2 fold increase is indicated. (b) Schematic representation of Smad2 (K19R, K20R, K39R and K19Q, K20Q, K39Q) acetylation mutants. (c) Schematic representation of Sirt1H363Y nucleotide mutation. Nucleotide and amino acid mutations are highlighted in red. (PDF 219 kb)

Additional file 2: Figure S2. HEK293T and HaCaT cells show Sirt1 and Smad2 nuclear colocalization after TGFβ treatment. (a) HEK293T cells were treated with TGFR inhibitor SB-431542 overnight and exposed to TGFβ for the indicated times, then were immuno-labeled for Smad2 and Sirt1 proteins. Nuclei were revealed with Hoestch-33258. (b) HaCaT-GFP-Smad2 cells were treated with TGFβ for the indicated times, then immuno-labeled. Corresponding labeling: Smad2 (green); Sirt1 (red); nuclei (blue). (PDF 1268 kb)

Additional file 3: Figure S3. Sirt1 dependent downregulation of TGFβ induced transcription requires functional Sirt1 activity. (a) NIH3T3 cells transfected with the indicated plasmids were treated with TGFβ for 6 h and measured for luciferase activity. “FH1”: FoxH1 transcription factor. Asterisks denote significant differences: ****p < 0.0001. (b) The expression of siRNA1 and siRNA2 interfere with Sirt1 expression. Hep3B cells were transfected with pTer-GFP-siRNA1 or pTer-GFP-siRNA2. Transfected cells were immuno-labeled for Sirt1 (red) and detected for GFP (green). Nuclei were revealed with Hoestch-33258. (c) Hep3B cells were transfected with the indicated GFP plasmids and enriched by Fluorescence-Activated cell sorting. Sirt1 expression level was detected by Western Blot and quantified. Sirt1/ß-actin is indicated (lane 1–4 over lane 1). ß-actin: loading control. NT: non-transfected cells. (PDF 1313 kb)

## Abbreviations

BMP: Bone Morphogenetic Proteins
BSA: bovine serum albumin
ChIP: chromatin immunoprecipitation assay
co-Smad: common-mediator Smad
DMSO: Dimethyl sulfoxide
DNAP: DNA pull-down assay
EMT: epithelial-mesenchymal transition
FH1: FoxH1 transcription factor
GST: glutathione S-transferase
H: Histone
HDAC: histone deacetylases
His-tag: Histidine tag
IP: immunoprecipitation
i-Smad: inhibitory Smad
LR: linker domain
MH1: Mad Homology domain 1
MH2: Mad Homology domain 2
Mix: Mixer transcription factor
Mut: mutant
NAD +: Nicotinamide adenine dinucleotide
NC: not calculable
NT: not transfected
ORF: open Reading frame
PBS: Phosphate-buffered saline
Pol II: polymerase II
R-Smads: receptor-regulated Smads
RT: room temperature
SAD: Smad4 activation domain
SBR: Smad Binding Region
SCR: scramble sequence
SEM: Standard Error of Measurement
SiRNA: small interference RNA
TGF-β: transforming growth factor beta
TGFβ-R: TFGβ receptors
TSA: Trichostatin-A
TSS: Transcription start site
WT: wild type

## References

[1] ten Dijke P, Hill CS (2004). New insights into TGF-beta-Smad signalling. Trends Biochem Sci.

[2] Blobe GC, Schiemann WP, Lodish HF (2000). Role of transforming growth factor beta in human disease. N Engl J Med.

[3] Macias MJ, Martin-Malpartida P, Massague J (2015). Structural determinants of Smad function in TGF-beta signaling. Trends Biochem Sci.

[4] Roberts AB (2002). The ever-increasing complexity of TGF-beta signaling. Cytokine Growth Factor Rev.

[5] Nicolas FJ, Hill CS (2003). Attenuation of the TGF-beta-Smad signaling pathway in pancreatic tumor cells confers resistance to TGF-beta-induced growth arrest. Oncogene.

[6] Siegel PM, Massague J (2003). Cytostatic and apoptotic actions of TGF-beta in homeostasis and cancer. Nat Rev Cancer.

[7] Ross S, Hill CS (2008). How the Smads regulate transcription. Int J Biochem Cell Biol.

[8] Wrighton KH, Feng XH (2008). To (TGF)beta or not to (TGF)beta: fine-tuning of Smad signaling via post-translational modifications. Cell Signal.

[9] Simonsson M, Kanduri M, Gronroos E, Heldin CH, Ericsson J (2006). The DNA binding activities of Smad2 and Smad3 are regulated by coactivator-mediated acetylation. J Biol Chem.

[10] Inoue Y, Itoh Y, Abe K, Okamoto T, Daitoku H, Fukamizu A, Onozaki K, Hayashi H (2007). Smad3 is acetylated by p300/CBP to regulate its transactivation activity. Oncogene.

[11] Tu AW, Luo K (2007). Acetylation of Smad2 by the co-activator p300 regulates activin and transforming growth factor beta response. J Biol Chem.

[12] Gronroos E, Hellman U, Heldin CH, Ericsson J (2002). Control of Smad7 stability by competition between acetylation and ubiquitination. Mol Cell.

[13] Simonsson M, Heldin CH, Ericsson J, Gronroos E (2005). The balance between acetylation and deacetylation controls Smad7 stability. J Biol Chem.

[14] Kume S, Haneda M, Kanasaki K, Sugimoto T, Araki S, Isshiki K, Isono M, Uzu T, Guarente L, Kashiwagi A, Koya D (2007). SIRT1 inhibits transforming growth factor beta-induced apoptosis in glomerular mesangial cells via Smad7 deacetylation. J Biol Chem.

[15] Buler M, Andersson U, Hakkola J (2016). Who watches the watchmen? Regulation of the expression and activity of sirtuins. FASEB J.

[16] Michan S, Sinclair D (2007). Sirtuins in mammals: insights into their biological function. Biochem J.

[17] Rajendran R, Garva R, Krstic-Demonacos M, Demonacos C (2011). Sirtuins: molecular traffic lights in the crossroad of oxidative stress, chromatin remodeling, and transcription. J Biomed Biotechnol.

[18] Vaquero A, Scher M, Lee D, Erdjument-Bromage H, Tempst P, Reinberg D (2004). Human SirT1 interacts with histone H1 and promotes formation of facultative heterochromatin. Mol Cell.

[19] Lavu S, Boss O, Elliott PJ, Lambert PD (2008). Sirtuins--novel therapeutic targets to treat age-associated diseases. Nat Rev Drug Discov.

[20] Haigis MC, Sinclair DA (2010). Mammalian sirtuins: biological insights and disease relevance. Annu Rev Pathol.

[21] Stunkel W, Campbell RM (2011). Sirtuin 1 (SIRT1): the misunderstood HDAC. J Biomol Screen.

[22] Li J, Qu X, Ricardo SD, Bertram JF, Nikolic-Paterson DJ (2010). Resveratrol inhibits renal fibrosis in the obstructed kidney: potential role in deacetylation of Smad3. Am J Pathol.

[23] Nakagawa T, Guarente L (2011). Sirtuins at a glance. J Cell Sci.

[24] Simmons GE, Pruitt WM, Pruitt K (2015). Diverse roles of SIRT1 in cancer biology and lipid metabolism. Int J Mol Sci.

[25] Byles V, Zhu L, Lovaas JD, Chmilewski LK, Wang J, Faller DV, Dai Y (2012). SIRT1 induces EMT by cooperating with EMT transcription factors and enhances prostate cancer cell migration and metastasis. Oncogene.

[26] Simic P, Williams EO, Bell EL, Gong JJ, Bonkowski M, Guarente L (2013). SIRT1 suppresses the epithelial-to-mesenchymal transition in cancer metastasis and organ fibrosis. Cell Rep.

[27] Chen IC, Chiang WF, Huang HH, Chen PF, Shen YY, Chiang HC (2014). Role of SIRT1 in regulation of epithelial-to-mesenchymal transition in oral squamous cell carcinoma metastasis. Mol Cancer.

[28] Heldin CH, Landstrom M, Moustakas A (2009). Mechanism of TGF-beta signaling to growth arrest, apoptosis, and epithelial-mesenchymal transition. Curr Opin Cell Biol.

[29] Boukamp P, Petrussevska RT, Breitkreutz D, Hornung J, Markham A, Fusenig NE (1988). Normal keratinization in a spontaneously immortalized aneuploid human keratinocyte cell line. J Cell Biol.

[30] Perez Oliva AB, Fernendez LP, Detorre C, Herraiz C, Martinez-Escribano JA, Benitez J, Lozano Teruel JA, Garcia-Borron JC, Jimenez-Cervantes C, Ribas G (2009). Identification and functional analysis of novel variants of the human melanocortin 1 receptor found in melanoma patients. Hum Mutat.

[31] Pierreux CE, Nicolas FJ, Hill CS (2000). Transforming growth factor beta-independent shuttling of Smad4 between the cytoplasm and nucleus. Mol Cell Biol.

[32] Nicolas FJ, De Bosscher K, Schmierer B, Hill CS (2004). Analysis of Smad nucleocytoplasmic shuttling in living cells. J Cell Sci.

[33] Germain S, Howell M, Esslemont GM, Hill CS (2000). Homeodomain and winged-helix transcription factors recruit activated Smads to distinct promoter elements via a common Smad interaction motif. Genes Dev.

[34] Vaziri H, Dessain SK, Ng Eaton E, Imai SI, Frye RA, Pandita TK, Guarente L, Weinberg RA (2001). hSIR2(SIRT1) functions as an NAD-dependent p53 deacetylase. Cell.

[35] Wong C, Rougier-Chapman EM, Frederick JP, Datto MB, Liberati NT, Li JM, Wang X (1999). Smad3-Smad4 and AP-1 complexes synergize in transcriptional activation of the c-Jun promoter byTransforming growth factor b. Mol Cell Biol.

[36] Martinez-Mora C, Mrowiec A, Garcia-Vizcaino EM, Alcaraz A, Cenis JL, Nicolas FJ (2012). Fibroin and sericin from Bombyx Mori silk stimulate cell migration through upregulation and phosphorylation of c-Jun. PLoS One.

[37] Alcaraz A, Mrowiec A, Insausti CL, Bernabe-Garcia A, Garcia-Vizcaino EM, Lopez-Martinez MC, Monfort A, Izeta A, Moraleda JM, Castellanos G, Nicolas FJ (2015). Amniotic membrane modifies the genetic program induced by TGFss, stimulating Keratinocyte proliferation and migration in chronic wounds. PLoS One.

[38] Hata A, Seoane J, Lagna G, Montalvo E, Hemmati-Brivanlou A, Massague J (2000). OAZ uses distinct DNA- and protein-binding zinc fingers in separate BMP-Smad and Olf signaling pathways. Cell.

[39] Levy L, Howell M, Das D, Harkin S, Episkopou V, Hill CS (2007). Arkadia activates Smad3/Smad4-dependent transcription by triggering signal-induced SnoN degradation. Mol Cell Biol.

[40] van de Wetering M, Oving I, Muncan V, Pon Fong MT, Brantjes H, van Leenen D, Holstege FC, Brummelkamp TR, Agami R, Clevers H (2003). Specific inhibition of gene expression using a stably integrated, inducible small-interfering-RNA vector. EMBO Rep.

[41] Inman GJ, Nicolas FJ, Hill CS (2002). Nucleocytoplasmic shuttling of Smads 2, 3, and 4 permits sensing of TGF-beta receptor activity. Mol Cell.

[42] Michishita E, Park JY, Burneskis JM, Barrett JC, Horikawa I (2005). Evolutionarily conserved and nonconserved cellular localizations and functions of human SIRT proteins. Mol Biol Cell.

[43] Tanno M, Sakamoto J, Miura T, Shimamoto K, Horio Y (2007). Nucleocytoplasmic shuttling of the NAD+−dependent histone deacetylase SIRT1. J Biol Chem.

[44] Watabe T, Kim S, Candia A, Rothbacher U, Hashimoto C, Inoue K, Cho KW (1995). Molecular mechanisms of Spemann's organizer formation: conserved growth factor synergy between Xenopus and mouse. Genes Dev.

[45] Candia AF, Watabe T, Hawley SH, Onichtchouk D, Zhang Y, Derynck R, Niehrs C, Cho KW (1997). Cellular interpretation of multiple TGF-beta signals: intracellular antagonism between activin/BVg1 and BMP-2/4 signaling mediated by Smads. Development.

[46] Lin X, Liang M, Feng XH (2000). Smurf2 is a ubiquitin E3 ligase mediating proteasome-dependent degradation of Smad2 in transforming growth factor-beta signaling. J Biol Chem.

[47] Warner DR, Pisano MM, Roberts EA, Greene RM (2003). Identification of three novel Smad binding proteins involved in cell polarity. FEBS Lett.

[48] Colland F, Jacq X, Trouplin V, Mougin C, Groizeleau C, Hamburger A, Meil A, Wojcik J, Legrain P, Gauthier JM (2004). Functional proteomics mapping of a human signaling pathway. Genome Res.

[49] Wicks SJ, Haros K, Maillard M, Song L, Cohen RE, Dijke PT, Chantry A (2005). The deubiquitinating enzyme UCH37 interacts with Smads and regulates TGF-beta signalling. Oncogene.

[50] Brown KA, Ham AJ, Clark CN, Meller N, Law BK, Chytil A, Cheng N, Pietenpol JA, Moses HL (2008). Identification of novel Smad2 and Smad3 associated proteins in response to TGF-beta1. J Cell Biochem.

[51] Fields S, Sternglanz R (1994). The two-hybrid system: an assay for protein-protein interactions. Trends Genet.

[52] Allen JB, Walberg MW, Edwards MC, Elledge SJ (1995). Finding prospective partners in the library: the two-hybrid system and phage display find a match. Trends Biochem Sci.

[53] Lin F, Morrison JM, Wu W, Worman HJ (2005). MAN1, an integral protein of the inner nuclear membrane, binds Smad2 and Smad3 and antagonizes transforming growth factor-beta signaling. Hum Mol Genet.

[54] Waddell DS, Liberati NT, Guo X, Frederick JP, Wang XF (2004). Casein kinase Iepsilon plays a functional role in the transforming growth factor-beta signaling pathway. J Biol Chem.

[55] Zheng M, McKeown-Longo PJ (2002). Regulation of HEF1 expression and phosphorylation by TGF-beta 1 and cell adhesion. J Biol Chem.

[56] Suzuki Y, Tanigaki T, Heimer D, Wang W, Ross WG, Murphy GA, Sakai A, Sussman HH, Vu TH, Raffin TA (1994). TGF-beta 1 causes increased endothelial ICAM-1 expression and lung injury. J Appl Physiol.

[57] Feng XH, Derynck R (2005). Specificity and versatility in tgf-beta signaling through Smads. Annu Rev Cell Dev Biol.

[58] Massague J, Seoane J, Wotton D (2005). Smad transcription factors. Genes Dev.

[59] Imai S, Armstrong CM, Kaeberlein M, Guarente L (2000). Transcriptional silencing and longevity protein Sir2 is an NAD-dependent histone deacetylase. Nature.

[60] Solomon JM, Pasupuleti R, Xu L, McDonagh T, Curtis R, DiStefano PS, Huber LJ (2006). Inhibition of SIRT1 catalytic activity increases p53 acetylation but does not alter cell survival following DNA damage. Mol Cell Biol.

[61] Luo J, Su F, Chen D, Shiloh A, Gu W (2000). Deacetylation of p53 modulates its effect on cell growth and apoptosis. Nature.

[62] Luo J, Nikolaev AY, Imai S, Chen D, Su F, Shiloh A, Guarente L, Gu W (2001). Negative control of p53 by Sir2alpha promotes cell survival under stress. Cell.

[63] Hill CS (2009). Nucleocytoplasmic shuttling of Smad proteins. Cell Res.

[64] Pederson T (2001). Protein mobility within the nucleus--what are the right moves?. Cell.

[65] Kong S, Kim SJ, Sandal B, Lee SM, Gao B, Zhang DD, Fang D (2011). The type III histone deacetylase Sirt1 protein suppresses p300-mediated histone H3 lysine 56 acetylation at Bclaf1 promoter to inhibit T cell activation. J Biol Chem.

[66] Torres G, Frisella PD, Yousuf SJ, Sarwar S, Baldinger L, Zakhary SM, Leheste JR (2008). A ChIP-cloning approach linking SIRT1 to transcriptional modificationof DNA targets. BioTechniques.

[67] Ross S, Cheung E, Petrakis TG, Howell M, Kraus WL, Hill CS (2006). Smads orchestrate specific histone modifications and chromatin remodeling to activate transcription. EMBO J.

[68] Pan M, Yuan H, Brent M, Ding EC, Marmorstein R (2012). SIRT1 contains N- and C-terminal regions that potentiate deacetylase activity. J Biol Chem.

[69] Morikawa M, Koinuma D, Miyazono K, Heldin CH (2013). Genome-wide mechanisms of Smad binding. Oncogene.

[70] Chong PA, Ozdamar B, Wrana JL, Forman-Kay JD (2004). Disorder in a target for the smad2 mad homology 2 domain and its implications for binding and specificity. J Biol Chem.

[71] Inman GJ (2005). Linking Smads and transcriptional activation. Biochem J.

[72] Ghisays F, Brace CS, Yackly SM, Kwon HJ, Mills KF, Kashentseva E, Dmitriev IP, Curiel DT, Imai SI, Ellenberger T. The N-terminal domain of SIRT1 is a positive regulator of endogenous SIRT1-dependent Deacetylation and transcriptional outputs. Cell Rep. 2015;10(10):1665-73. 10.1016/j.celrep.2015.02.036 PMC4565781 25772354

[73] Davenport AM, Huber FM, Hoelz A (2014). Structural and functional analysis of human SIRT1. J Mol Biol.

[74] Kang MR, Lee SW, Um E, Kang HT, Hwang ES, Kim EJ, Um SJ (2010). Reciprocal roles of SIRT1 and SKIP in the regulation of RAR activity: implication in the retinoic acid-induced neuronal differentiation of P19 cells. Nucleic Acids Res.

[75] Folk P, Puta F, Skruzny M (2004). Transcriptional coregulator SNW/SKIP: the concealed tie of dissimilar pathways. Cell Mol Life Sci.

