# Supplementary figures and images for: Sirt1 interaction with active Smad2 modulates transforming growth factor-β regulated transcription
Source: Cell Commun Signal. 2017 Nov 29;15:50. doi: 10.1186/s12964-017-0205-y (PMC5706420; doi:10.1186/s12964-017-0205-y)

**a**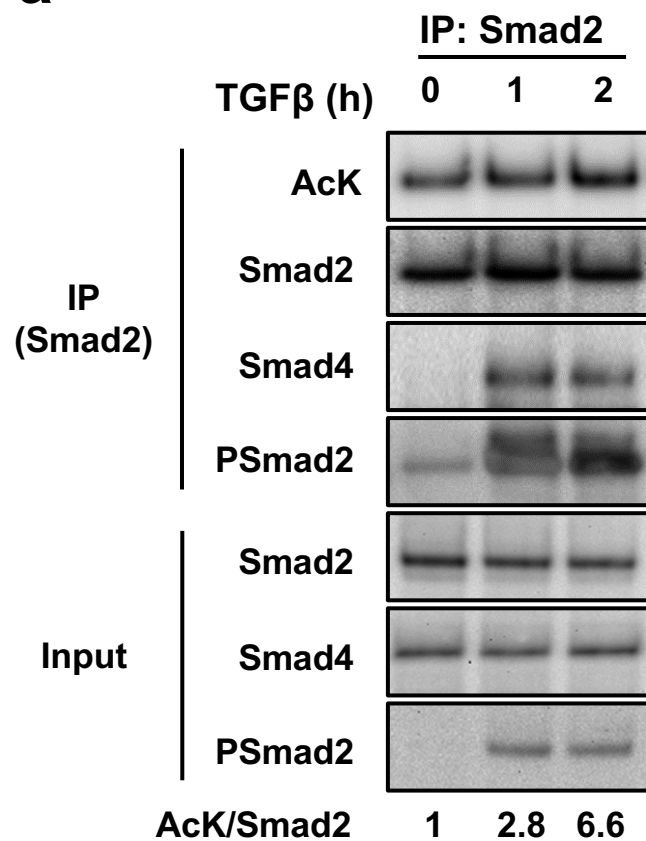**b**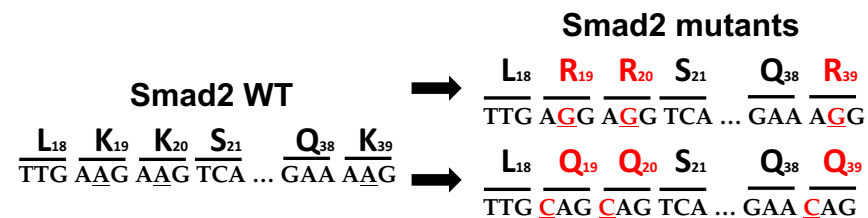**c**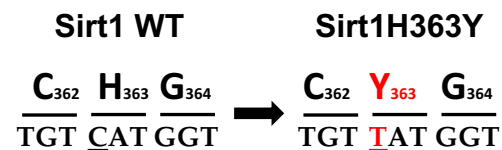

Supplement: Supplementary file 1 — TGFβ induces the acetylation of Smad2. (a) Hep3B cells were serum starved overnight, treated with TSA for 8 h followed by TGFβ for the indicated times. Smad2 was purified by immunoprecipitation, then acetyl-Lys,Smad2, Smad4 and PSmad2 were detected by Western Blot. Ack/Smad2 fold increase is indicated. (b) Schematic representation of Smad2 (K19R, K20R, K39R and K19Q, K20Q, K39Q) acetylation mutants. (c) Schematic representation of Sirt1H363Y nucleotide mutation. Nucleotide and amino acid mutations are highlighted in red. (PDF 219 kb) [file 12964_2017_205_MOESM1_ESM.pdf]

**a**

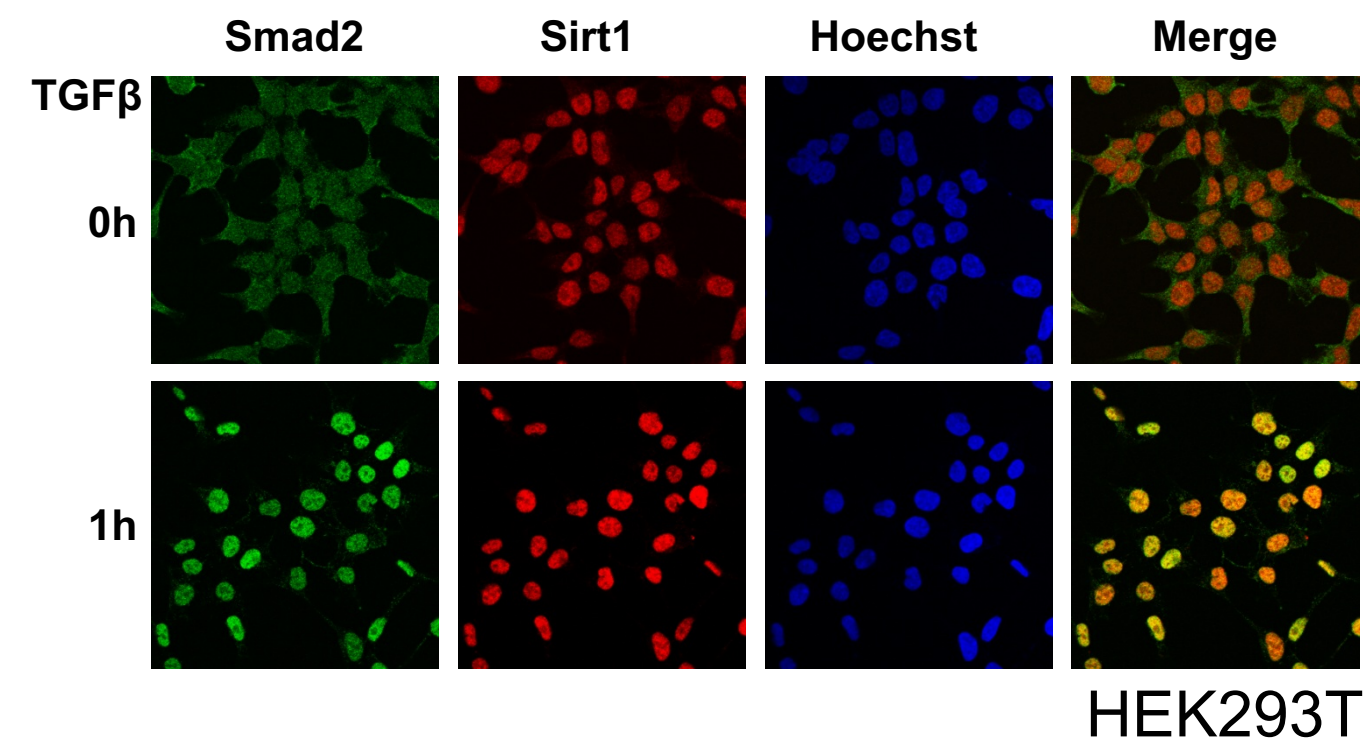

**b**

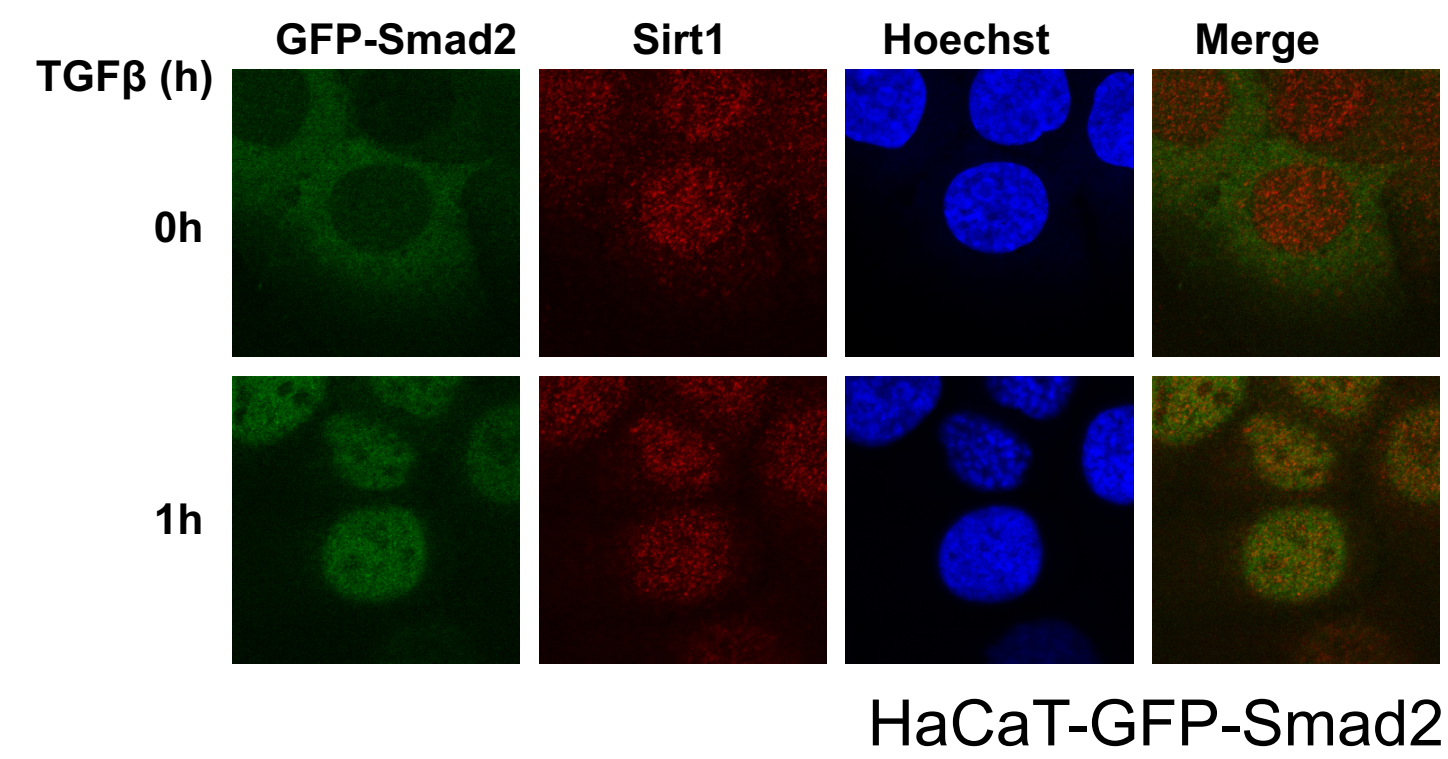

Supplement: Supplementary file 2 — HEK293T and HaCaT cells show Sirt1 and Smad2 nuclear colocalization after TGFβ treatment. (a) HEK293T cells were treated with TGFR inhibitor SB-431542 overnight and exposed to TGFβ for the indicated times, then were immuno-labeled for Smad2 and Sirt1 proteins. Nuclei were revealed with Hoestch-33258. (b) HaCaT-GFP-Smad2 cells were treated with TGFβ for the indicated times, then immuno-labeled. Corresponding labeling: Smad2 (green); Sirt1 (red); nuclei (blue). (PDF 1268 kb) [file 12964_2017_205_MOESM2_ESM.pdf]

**a**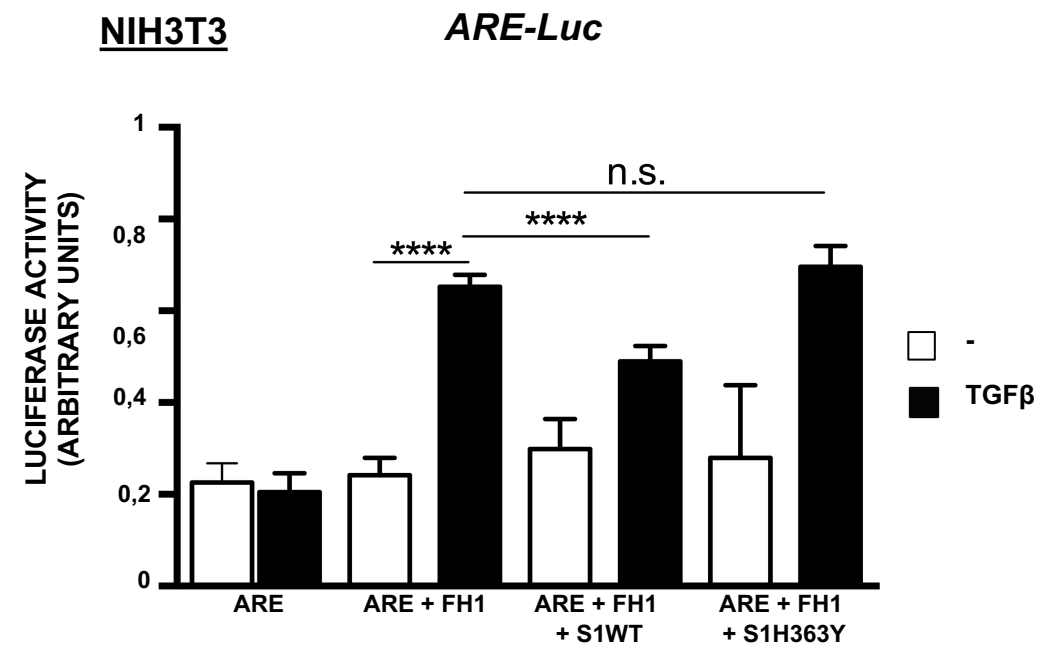**b**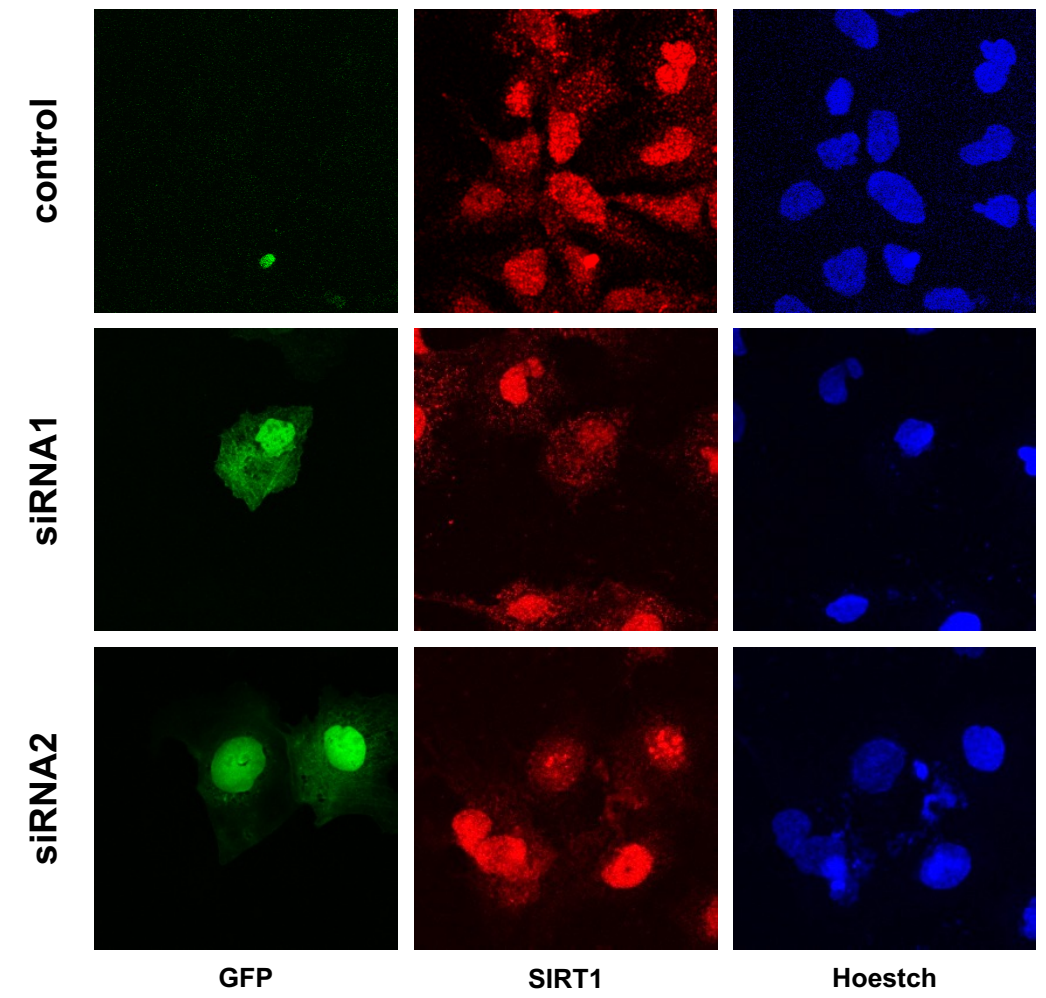**c**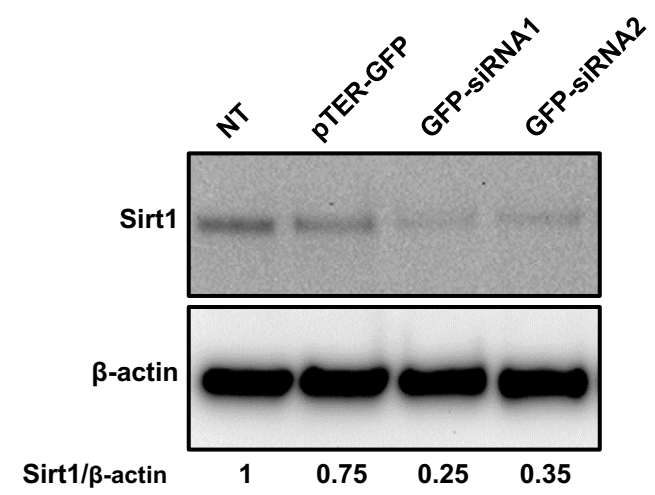

Supplement: Supplementary file 3 — Sirt1 dependent downregulation of TGFβ induced transcription requires functional Sirt1 activity. (a) NIH3T3 cells transfected with the indicated plasmids were treated with TGFβ for 6 h and measured for luciferase activity. “FH1”: FoxH1 transcription factor. Asterisks denote significant differences: ****p < 0.0001. (b) The expression of siRNA1 and siRNA2 interfere with Sirt1 expression. Hep3B cells were transfected with pTer-GFP-siRNA1 or pTer-GFP-siRNA2. Transfected cells were immuno-labeled for Sirt1 (red) and detected for GFP (green). Nuclei were revealed with Hoestch-33258. (c) Hep3B cells were transfected with the indicated GFP plasmids and enriched by Fluorescence-Activated cell sorting. Sirt1 expression level was detected by Western Blot and quantified. Sirt1/ß-actin is indicated (lane 1–4 over lane 1). ß-actin: loading control. NT: non-transfected cells. (PDF 1313 kb) [file 12964_2017_205_MOESM3_ESM.pdf]
